# Membrane-proximal TRAIL species are incapable of inducing short circuit apoptosis signaling: Implications for drug development and basic cytokine biology

**DOI:** 10.1038/srep22661

**Published:** 2016-03-03

**Authors:** Katharina Tatzel, Lindsay Kuroki, Igor Dmitriev, Elena Kashentseva, David T. Curiel, S. Peter Goedegebuure, Matthew A. Powell, David G. Mutch, William G. Hawkins, Dirk Spitzer

**Affiliations:** 1 Washington University School of Medicine, Department of Surgery, 660 S. Euclid Avenue, St. Louis, Missouri 63110, USA; 2 Washington University School of Medicine, Division of Gynecologic Oncology, 660 S. Euclid Avenue, St. Louis, Missouri 63110, USA; 3 Washington University School of Medicine, Department of Radiation Oncology, 660 S. Euclid Avenue, St. Louis, Missouri 63110, USA; 4 Alvin J. Siteman Cancer Center, 660 S. Euclid Avenue, St. Louis, Missouri 63110, USA

## Abstract

TRAIL continues to garner substantial interest as a recombinant cancer therapeutic while the native cytokine itself serves important tumor surveillance functions when expressed in membrane-anchored form on activated immune effector cells. We have recently developed the genetically stabilized TRAIL platform TR3 in efforts to improve the limitations associated with currently available drug variants. While in the process of characterizing mesothelin-targeted TR3 variants using a single chain antibody (scFv) delivery format (SS-TR3), we discovered that the membrane-tethered cytokine had a substantially increased activity profile compared to non-targeted TR3. However, cell death proceeded exclusively via a bystander mechanism and protected the mesothelin-positive targets from apoptosis rather than leading to their elimination. Incorporation of a spacer-into the mesothelin surface antigen or the cancer drug itself-converted SS-TR3 into a cis-acting phenotype. Further experiments with membrane-anchored TR3 variants and the native cytokine confirmed our hypothesis that membrane-proximal TRAIL species lack the capacity to physically engage their cognate receptors coexpressed on the same cell membrane. Our findings not only provide an explanation for the “peaceful” coexistence of ligand and receptor of a representative member of the TNF superfamily but give us vital clues for the design of activity-enhanced TR3-based cancer therapeutics.

Apoptosis is an evolutionarily well-conserved process for the coordinated removal of undesired cells from a multicellular organism. As such, it serves important functions ranging from early embryologic development to the eradication of senescent and potentially cancerous cells throughout our lives^1^,^2^. Members of the tumor-necrosis factor (TNF) superfamily are critically involved in these processes and share several common features, including ligand trimerization, type-II transmembrane anchorage and systemic availability following proteolytic cleavage from the cell surface^3^,^4^. One particular member of this family, TNF-related apoptosis-inducing ligand (TRAIL) interacts with five endogenous receptors, four of which are cell membrane associated (DR4, DR5, DcR1, DcR2), whereas the fifth receptor, osteoprotegerin (OPG) constitutes a fluid phase receptor^5^.

Endogenous and recombinant TRAIL require trimerization in order to gain functional activity. Among the four classes of TNF family members, TRAIL is unique in that it contains an unpaired cysteine per protomer (3 sulfhydryl groups/trimer), which has to be kept in a reduced state in order for the trimer to be biologically active. Attempts to produce bioactive, soluble TRAIL from monomeric cDNAs in mammalian cells have failed due to intermolecular disulfide bridge formation^6^. This limitation prompted us to combine the three TRAIL protomers into a single, head-to-tail fusion protein (TR3), to achieve increased stability and flexibility with regard to downstream functionalization efforts, e.g. for the design of biomarker-targeted TR3 variants via modular domain exchange under strict stoichiometric control^7^,^8^.

Since its discovery, recombinant soluble TRAIL has received much attention for its ability to destroy cancer cells and has since been explored in a number of clinical trials^9^,^10^,^11^. Interestingly, we and others have shown that tethering soluble TRAIL to the cancer cells substantially enhances its bioactivity^7^,^12^,^13^. For example, membrane tethering of MUC16-targeted Meso-TR3 to ovarian cancer cells was capable of overriding the therapeutic plateau of non-targeted TR3 (ref. 7) caused by an overexpression of the prosuvival factor cFLIP^14^.

Here, we built on our earlier studies and designed TR3 variants targeted to mesothelin, a tumor biomarker frequently overexpressed in a number of human malignancies, including pancreatic cancer, ovarian cancer and mesothelioma^15^,^16^,^17^,^18^,^19^. The targeting strategy was based on the mesothelin-specific single chain antibody (scFv) SS^20^, which was genetically fused to the amino-terminus of the TR3 drug platform. During the initial characterization phase of our newly developed drug candidates, we discovered that the overall potency of targeted SS-TR3 was indeed much increased in the presence of mesothelin expression. Paradoxically, the mesothelin-positive targets were unexpectedly protected from cell death and were actively enriched following drug exposure. Further investigations confirmed a pivotal role of a spacer domain provided either in *“cis”* (built into the targeted cancer drug itself) or in *“trans”* (incorporated into the surface-expressed target antigen), which had a profound effect on the mechanism of cancer cell death.

The inability to induce cell death of mesothelin-expressing tumor cells directly with spacer-deficient SS-TR3 prompted the question if the TR3 domain of the fusion protein was in fact capable of physically engaging the death receptors located on the same membrane. Along these lines, a similar scenario in which native, membrane TRAIL is coexpressed along with several of its death receptors has been demonstrated in natural killer (NK) and cytotoxic CD8 + T cells^21^, raising the possibility of autocrine-like death receptor signaling events and the potential threat of immune effector cell elimination^22^,^23^. Even though the authors convincingly demonstrated protection of these cells from TRAIL-mediated cell death via heightened cFLIP expression^21^, the ability of membrane TRAIL to physically interact with its own receptors on the same cell surface to induce short circuit death receptor signaling was not addressed in this study.

In an attempt to interrogate if the native, membrane-bound cytokine and other membrane-proximal TRAIL species were indeed capable of binding their cognate receptors when coexpressed on the same cell membrane, we generated membrane-anchored TRAIL forms (native and TR3-based) and studied the ligand/receptor interactions by flow cytometry on a single cell level. Our results confirmed that membrane-proximal TRAIL species were incapable of interacting with their own receptors due to steric constrains. Overall, we gained important insight into the spatial requirements for a functional interaction between TRAIL and DR5 at the plasma membrane, not only for the informed design of improved TR3-based cancer drugs but also with regard to the biology of a native and representative member of the large TNF superfamily.

## Results

### Biomarker-targeted TR3 has a superior activity profile on mesothelin-positive cancer cells

We have previously reported on TR3 fusion proteins tethered to mouse red blood cells (RBC) using the single chain antibody fragment Ter119 (scFv-Ter119), essentially converting soluble biomolecules into membrane-bound counterparts [Fig. S1a and refs 8,24]. The TR3-decorated RBC nanoparticles acted as potent effector cells capable of killing TRAIL-sensitive Jurkat cells. Importantly, incorporation of an elongated and flexible spacer between the scFv-Ter119 targeting and the TR3 effector domain resulted in a superior activity profile (Fig. S1b,c, Ter119-S-TR3). The molecular spacer used to separate the scFv targeting domain from the TR3 effector domain is comprised of four short consensus repeats (SCRs), ~60 amino acid globular and flexible domains conserved among complement regulatory proteins (CRPs). The particular spacer used in our targeted TR3 therapeutics is comprised of SCR1 of human decay-accelerating factor (DAF, CD55) and SCRs 15–17 of human complement receptor 1 (CR1, CD35) and has been described earlier^8^. Human DAF is a GPI-anchored glycoprotein with a molecular mass of 70 kDa and contains four SCRs and an elongated stalk region^25^. CR1 is a transmembrane-anchored glycoprotein with a molecular mass of ~200 kDa and contains 28 SCRs^26^. We refer the readers to additional articles for a more detailed information about the structural and functional features of DAF and CR1^27^,^28^,^29^,^30^,^31^. On a mechanistic level, apoptosis induction required cell-cell contact (bystander mechanism) and was efficiently blocked via spatial separation of target and effector cells (Fig. S1d). The key advantage of studying TR3 drug targeting with RBCs as a model system is that these cells do not express death receptors on their membrane, which eliminates binding events mediated by TR3/DR interactions on the RBC membrane. Not surprisingly, this situation becomes more complex when dual-domain therapeutics, such as scFv-TR3, are targeted to biomarker-positive cancer cells, which also express various TRAIL receptors.

**Figure 1:**
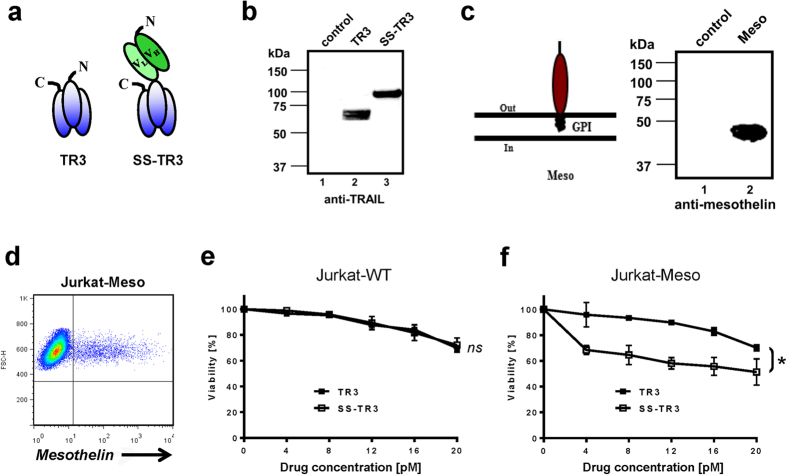
Mesothelin-targeting increases the bioactivity of soluble TR3. (**a**) Schematic representation of recombinant TR3 forms. The functional domain of secreted wild-type TRAIL has been genetically linked, resulting in the TRAIL-based drug platform TR3 (left). TR3 was subsequently fused at its N-terminus with scFv-SS, resulting in the mesothelin-targeted TR3 variant SS-TR3 (right). (**b**) Western blot analysis of recombinant TR3 forms produced in HEK293T cells (anti-TRAIL pAb). Supernatant from mock transfected cells was used as a control (lane 1). (**c**) Schematic representation of FLAG-tagged, mature human mesothelin inserted into the membrane via a glycosylphosphatidyl (GPI) anchor. Western blot analysis of Jurkat cells infected with a retroviral vector encoding FLAG-tagged human mesothelin (~40 kDa). (**d**) Anti-FLAG immunostaining confirms expression of the biomarker on Jurkat cells using flow cytometry. (**e**) Jurkat-WT and (**f**) ~5% Jurkat-Meso cells were treated with increasing concentrations of TR3 and SS-TR3 and cell viability was determined (n = 3, mean ± SEM; ns, not significant, *P < 0.025, *t-test*).

In an effort to develop the TR3-based drug technology further, we aimed at targeting soluble TR3 directly to the cancer biomarker mesothelin. As a targeting vehicle, we employed a mesothelin-specific antibody fragment [scFv-SS, ref. 20], validated using ectopically mesothelin-expressing HEK293T cells (Fig. S2). In order to generate mesothelin-targeted TR3, scFv-SS was placed at the amino-terminus of soluble TR3 (Fig. 1a, SS-TR3). A corresponding spacer-containing variant was generated for comparative studies (Fig. S3a, SS-S-TR3). The recombinant proteins were produced in HEK293T cells and the single chain character of the fusion proteins were verified by Western blot analysis (Fig. 1b and Fig. S3b). Jurkat cells served as a model system because they only express the activating death receptor DR5 and lack endogenous mesothelin expression, which was introduced utilizing retroviral gene transfer (Jurkat-Meso) (Fig. 1c,d).

As a means to study the potential impact of the targeting domain (scFv-SS) on death receptor recognition, the activity profiles of parental TR3 and targeted SS-TR3 were first evaluated on wild-type Jurkat cells (Jurkat-WT). At equimolar concentrations, both drugs induced cell death in a dose-dependent fashion and, most importantly, with equivalent potency (Fig. 1e). This phenotype suggests that the scFv targeting moiety of SS-TR3 does not interfere with apoptosis signaling and allows unrestricted access of the TR3 effector moiety of the dual-domain fusion protein to the death receptors (DR5). When the same drugs were tested on a ~5% mesothelin-containing Jurkat cell pool (Jurkat-Meso, same as in Fig. 1d), a substantial increase in cell death was noted only for SS-TR3, but not for parental TR3 (Fig. 1f). This enhanced cell death induction required mesothelin binding, i.e. membrane tethering, since blocking experiments with scFv-SS resulted in a dose-dependent reduction of apoptosis (Fig. S3c). Importantly, ectopic mesothelin expression did not alter the sensitivity of Jurkat-Meso cells to TRAIL, since exposure of Jurkat-WT cells to non-targeted TR3 resulted in similar activity profiles (Fig. 1e,f).

### Biomarker-targeted TR3 induces cell death via bystander mechanism and protects mesothelin-positive targets from apoptosis

**Figure 2:**
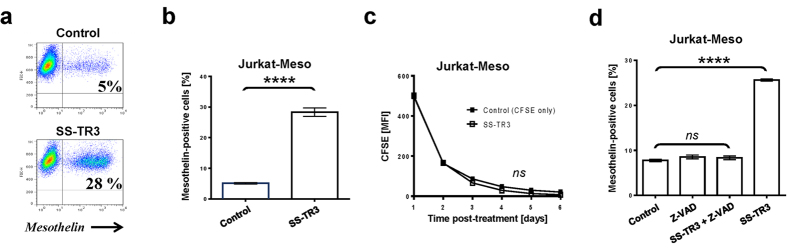
Mesothelin-targeted TR3 protects its target cells from cell death. (**a**) Representative example of Jurkat-Meso cell enrichment following SS-TR3 drug exposure. The cells were treated with SS-TR3 for six days or with medium alone (ctrl), followed by assessing the percentage of mesothelin-positive cells by flow cytometry. (**b**) Graphic representation of the data shown in (**a**) (n = 3, mean ± SEM; ****P < 0.0001, *t-test*). (**c**) Jurkat-Meso cells (~8%) do not acquire a growth advantage following treatment with SS-TR3 (CFSE dilution assay without apoptosis blockade) (n = 3, mean ± SEM; ns, not significant, *t-test*). (**d**) Jurkat-Meso cells do not expand following SS-TR3 treatment in the presence of pan-caspase inhibition (Z-VAD-FMK) (n = 3, mean ± SEM; ns, not significant, ****P < 0.0001, *one-way ANOVA*).

Since the overall killing activity of mesothelin-targeted SS-TR3 was consistently and noticeably higher than the percentage of mesothelin-expressing targets within the cell pools (Fig. 1f), we performed flow cytometry to determine if the Jurkat-Meso cells were indeed eliminated by SS-TR3. In contrast to our expectations, we noticed a sharp increase in the ratio of mesothelin-positive cells from 5% to 28%, following a six day recovery phase post-treatment (Fig. 2a,b). Depending on the experimental conditions, these numbers increased to nearly 90% (Fig. S4a). Conversely, when the same starting population of mesothelin-positive cells (5%) was treated with spacer-containing SS-S-TR3, we detected a reduction in the percentage of mesothelin-positive cells within the cell pool (Fig. S4b).

From a drug development point of view, the latter outcome represented a more desired feature, given that the initial goal was to selectively eradicate the mesothelin-positive cancer cells from a mixed pool of cells. Based on these results, however, we concluded that the mesothelin-expressing cells must have been protected from SS-TR3-induced cell death and served primarily as effector surfaces, killing preferentially mesothelin-negative bystander cells (Jurkat-WT), in analogy to the RBC-based nanoparticle concept referred to above [Fig. S1 and ref. 8].

To investigate the nature of this unanticipated phenomenon in greater detail, we had to rule out that mesothelin engagement by SS-TR3 unintentionally increased the proliferative capacity of Jurkat-Meso cells, leading to their rapid expansion in a mix with Jurkat-WT cells. Using a CFSE-dilution assay, we monitored the proliferative capacity of an entire cell pool containing ~8% Jurkat-Meso cells followed by SS-TR3 drug treatment in the absence of apoptosis blockade. The CFSE signal intensities homogeneously decreased over a six-day period with similar kinetics found in SS-TR3 and non-treated control cells (Fig. 2c). These results were indicative of a lack of a growth advantage of mesothelin-expressing Jurkat cells following SS-TR3 tethering. In order to confirm this hypothesis, we performed SS-TR3 treatment under conditions of apoptosis blockade using the pan-caspase inhibitor Z-VAD-FMK. In the presence of SS-TR3 and apoptosis prevention (Z-VAD-FMK), the mesothelin-positive cell count remained unchanged relative to cells treated with medium or apoptosis inhibitor alone (Fig. 2d). Only when the caspase inhibitor was omitted, the ratio of SS-TR3-treated Jurkat-Meso cells expanded again as predicted and served as a positive control. Based on these results, we concluded that the mesothelin-expressing cells were indeed protected from SS-TR3-induced cell death and served exclusively as effector surfaces, inducing cell death only on mesothelin-negative bystander cells (Jurkat-WT), similar in nature to the RBC-based nanoparticle concept reported earlier^8^.

### Spatial configurations of the target antigen determine the killing mechanism of targeted TR3 biologics

**Figure 3:**
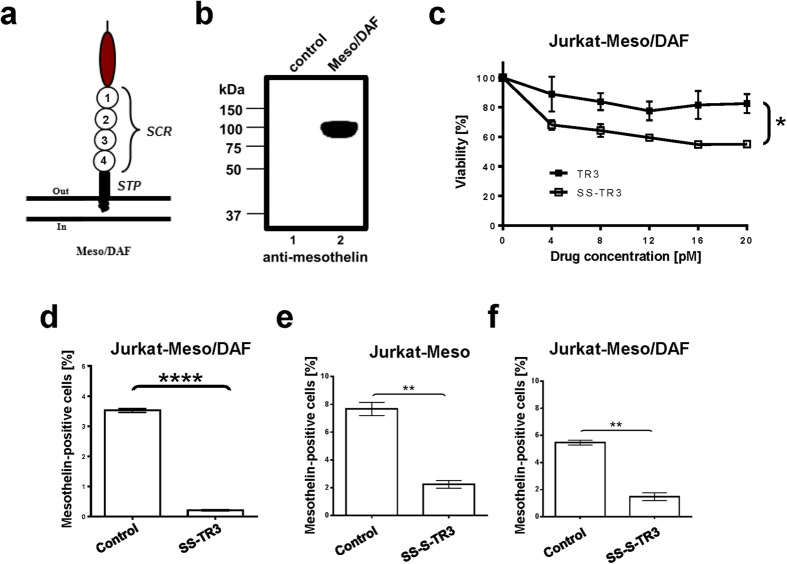
Spatial determinants define the phenotypic properties of mesothelin-targeted TR3. (**a**) Schematic representation of the Meso/DAF fusion protein. The GPI-anchored human decay-accelerating factor (DAF) was used to physically elevate the mesothelin binding site of scFv-SS away from the plasma membrane. The short consensus repeats (SCRs) of DAF are indicated (1–4). (**b**) Western blot analysis of Jurkat cells stably expressing the mesothelin/DAF fusion protein (Meso/DAF, ~110 kDa). (**c**) Jurkat-Meso/DAF cells (~5%) were treated with increasing concentrations of TR3 and SS-TR3 and cell viability was determined. (n = 3, mean ± SEM; *P < 0.027, *t-test*). (**d**) Meso/DAF-expressing Jurkat cells are eliminated from the cell pool following SS-TR3 treatment (n = 6, mean ± SEM; ****P < 0.0001, *t-test*). (**e**) Jurkat-Meso (~8%) and (**f**) Jurkat-Meso/DAF cells (~6%) were treated with SS-S-TR3. The consequence of drug treatment on the ratio of mesothelin-positive cells was assessed by flow cytometry. Please note that the mesothelin-positive cell population decreased in both instances (n = 3, mean ± SEM; **P = 0.0017 [Jurkat-Meso], **P = 0.0021 [Jurkat-Meso/DAF], *t-test*).

We hypothesized that the close proximity to the plasma membrane of spacer-deficient SS-TR3, when bound to mesothelin, prevented a direct interaction with death receptors located on the same plasma cell membrane, while the spacer-containing SS-S-TR3 could engage these receptors, resulting in target cell elimination. If this assumption was correct, we expected to restore target cell killing with SS-TR3 by incorporating the spacer domain of SS-S-TR3 into the mature, membrane-anchored form of human mesothelin, thereby providing the spacer in a “trans” configuration relative to spacer-deficient SS-TR3. To test this prediction, we generated a fusion protein in which the GPI-anchored decay-accelerating factor (DAF) served as a spacer to elevate the mesothelin binding site of SS-TR3 further away from the plasma membrane (Fig. 3a, Meso/DAF). We subsequently introduced this construct into Jurkat cells via retroviral infection and confirmed its identity by Western blot analysis (Fig. 3b, Jurkat-Meso/DAF). Functional experiments with non-targeted TR3 and SS-TR3 on Jurkat-Meso/DAF cells resulted in activity profiles similar to those observed earlier with Jurkat-Meso cells (Fig. 3c and compare Fig. 1f). However, and in stark contrast to the effects seen with Jurkat-Meso cells, SS-TR3 treatment resulted in elimination of Jurkat-Meso/DAF cells from the cell pool (Fig. 3d). These results were phenotypically identical to the treatment of Jurkat-Meso cells with spacer-containing SS-S-TR3 (Fig. 3e). We concluded from these data that SS-TR3 was able to engage in extrinsic death pathway activation on mesothelin-expressing targets only when the mesothelin binding epitope was located further away from the plasma membrane. This configuration allowed the membrane-tethered cytokine to bend over and engage its receptors in a productive fashion (cis-acting mechanism), similar to what spacer-containing SS-S-TR3 could achieve in both Jurkat-Meso and Jurkat-Meso/DAF cells (Fig. 3f).

### Membrane-proximal TRAIL species cannot engage their death receptors located on the same cell surface due to steric constraints

We have demonstrated that SS-TR3, when bound to mesothelin-expressing targets was only capable of inducing bystander cell death (trans-acting mechanism), while cis-apoptosis induction was not observed using a stringent model of highly TRAIL-sensitive Jurkat reporter cells. These results implied that SS-TR3 was incapable of engaging death receptors located on mesothelin-positive cancer cells. As such, mesothelin-bound SS-TR3 was viewed as a membrane-anchored form of TR3 (via attachment to GPI-anchored mesothelin). Here, we sought to demonstrate that mesothelin-bound SS-TR3 is phenotypically equivalent to membrane-anchored native TRAIL, thereby providing a link between targeted cancer drug development and native TRAIL biology.

**Figure 4:**
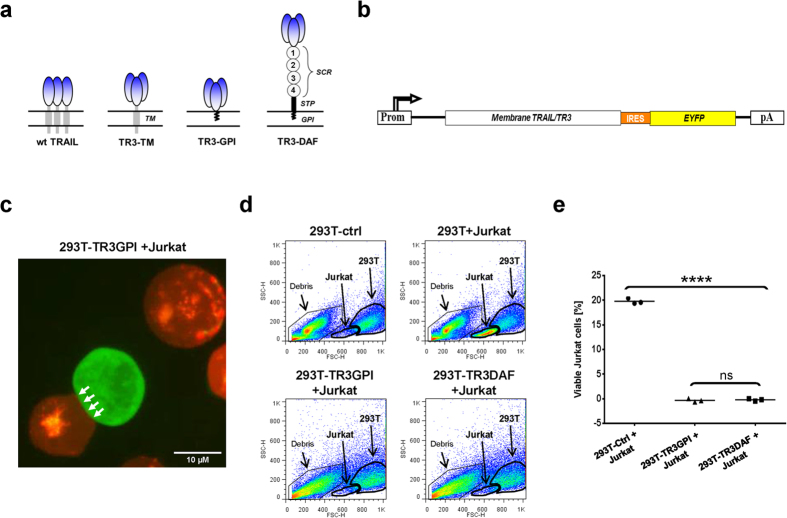
Membrane-anchored recombinant TR3 variants convert HEK293T cells into potent apoptosis-inducing effector cells. (**a**) Structural features of wild-type, native TRAIL (wt TRAIL) and recombinant TR3 forms anchored to the membrane via carboxyl-terminal incorporation of a VEGFR-2-derived transmembrane domain (TR3-TM), the GPI-encoding signal sequence of human DAF (TR3-GPI) or the entire mature form of human DAF (TR3-DAF). The short consensus repeats (SCRs) of DAF are indicated (1–4). Please note the distinct architectural differences between native TRAIL and the TR3 variants. In order for native TRAIL to be functionally active, three individual monomers have to join at the plasma membrane (1:1 stoichiometry), while each TR3 trimer associates with the membrane in a 3:1 stoichiometry (1 functional TR3 trimer/1 membrane anchor [TM or GPI]). (**b**) Schematic illustration of a bicistronic expression format^32^, in which the expression level of a membrane-anchored surface molecule is proportionally correlated with the expression pattern of the marker protein. (**c**) HEK293T cells were transiently transfected with TR3-DAF (green). The following day, the cells were washed and mixed with PKH26-labeled Jurkat target cells (red, E/T ratio 1:1.3) and allowed to sediment for 2 h at ambient temperature. The cells were then subjected to fluorescent microscopy. Please note the formation of heterotypic cell aggregates and smooth cell-cell junctions, indicative of tight interactions between TR3-DAF-expressing 293T cells and DR5-positive Jurkat cells. (**d**) Transiently transfected HEK293T cells were overlaid with Jurkat cells for 24 h, the mixtures were harvested and subjected to FACS analysis (FSC/SSC gating). Non-transfected HEK293T cells were used as a control. Please note that the viable Jurkat cells completely disappeared from their respective analysis gates following exposure to TR3-GPI- and TR3-DAF-expressing HEK293T cells. The “Debris” gate contains HEK293T cellular debris at baseline (upper panels) and additional debris originating from the “Jurkat” gates only under conditions in which HEK293T cells express the membrane-anchored TR3 variants TR3GPI and TR3DAF (lower panels). (**e**) Quantitative analysis of the data presented in (**d**) after background subtraction (n = 3, ****P < 0.0001; ns, not significant, *one-way ANOVA*).

In order to address this hypothesis, we developed an immune-based detection system in which the expression patterns of transmembrane- and GPI-anchored forms of the cytokine (Fig. 4a, wt TRAIL, TR3-TM, TR3-GPI and TR3-DAF) were intimately correlated with the expression profile of a marker protein (eYFP) using a bicistronic vector design [Fig. 4b and refs 32,33]. It is worth mentioning that the ability to anchor TR3 via its carboxyl-terminus to the plasma membrane has only become possible with this novel drug design, a scenario unlikely to be compatible with traditional, monomer-based TRAIL expression systems. Initial expression analyses suggested that the membrane-anchored TR3 variants were folded correctly, demonstrated by their ability not only to form heterologous cell aggregates with DR5-positive Jurkat cells (Fig. 4c, arrows) but also to induce their contact-dependent cell death (Fig. 4d,e). The assay principle to study ligand/receptor interactions via flow cytometry is exemplified with a signaling-deficient form of human vascular endothelial growth factor receptor-2 (VEGFR-2, KDRdCYT-eYFP) expressed in cells naturally lacking membrane-anchored forms of its ligands (Fig. S5a). Here, a proportional relationship between YFP and the KDR surface antigen documents an unrestricted accessibility of the anti-KDR mAb to its antigen over the entire expression range (Fig. S5b, diagonal arrow).

**Figure 5:**
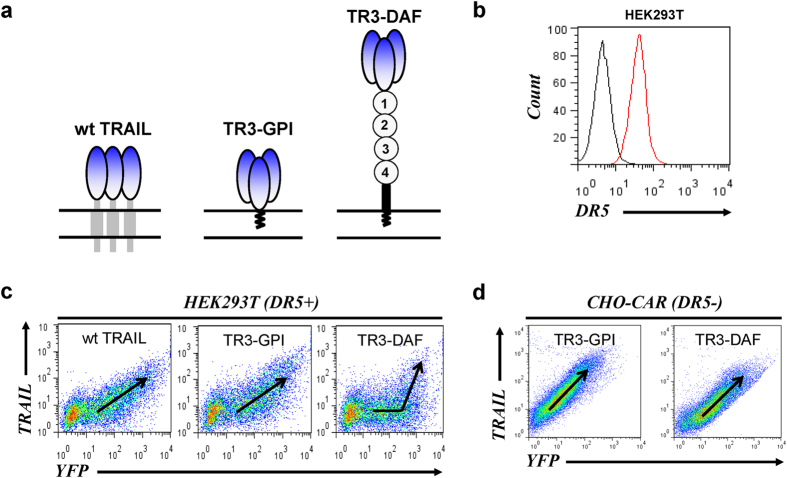
Membrane-proximal TRAIL is unable to interact with its receptors on the same plasma cell membrane. (**a**) Schematic representation of the structural features of wild-type TRAIL, and the GPI-anchored TR3 forms TR3-GPI and TR3-DAF expressed from a bicistronic configuration using an eYFP marker. The short consensus repeats (SCRs) of DAF are indicated (1–4). (**b**) HEK293T cells express death receptor 5 (DR5). (**c**) HEK293T cells were transiently transfected with wt TRAIL, TR3-GPI and TR3-DAF and analyzed by flow cytometry using a blocking anti-TRAIL mAb. The expression profiles for wt TRAIL and TR3-GPI result in a diagonal pattern (proportional relationship between TRAIL and YFP marker), whereas TR3-DAF expression results in a lack of TRAIL detection in an area where DR5 expression was detected. (**d**) Chinese hamster ovary cells (DR5-), expressing human CAR receptor (CHO-CAR), were infected with adenoviral vectors containing TR3-GPI and TR3-DAF using the same bicistronic configuration described in (**a**) and analyzed for their TRAIL expression as described in (**c**). Both surface proteins produced similar expression profiles (proportional relationship between TRAIL and YFP marker) with TR3-DAF being 100% detectable over the entire YFP expression range, suggesting a lack of interference with a surface receptor for the TR3 domain of the fusion protein.

This concept was then used to assess the binding ability of membrane-anchored TRAIL ligands (Fig. 5a, wt TRAIL and TR3 variants) with DR5 employing a blocking anti-TRAIL mAb. It was predicted that when the ligand was tightly bound to the receptor on the cell surface, the binding epitope of the anti-TRAIL mAb would be masked and lead to a reduction in surface staining intensity. When wt TRAIL and TR3-GPI were expressed in DR5-positive HEK293T cells (Fig. 5b), the staining profiles revealed a proportional relationship between the surface antigens and the eYFP reporter (Fig. 5c, wt TRAIL and TR3-GPI, diagonal staining profiles). This result was of key importance, as it demonstrated a phenotypic similarity between the native cytokine and recombinant versions of our TR3-based membrane-anchored fusion proteins. Similar staining profiles were obtained with all spacer-deficient TRAIL variants used in our study, including TR3-TM (Fig. S6), phenotypically identical to the KDR/eYFP expression pattern (compare Fig. S5). In contrast, spacer-containing TR3-DAF remained eYFP single-positive, i.e. lacked TRAIL detection in the low-to-intermediate expression level range (Fig. 5c, TR3-DAF, horizontal part of the arrow) and only became double-positive at high expression levels (Fig. 5c, TR3-DAF, nearly vertical part of the arrow). These results are compatible with the hypothesis that membrane-proximal TRAIL variants (wt TRAIL, TR3-GPI and TR3-TM) are indeed incapable of interacting with their native receptor(s) on the cell surface and are therefore fully detectable using function-blocking TRAIL antibodies. Only when the TR3 domain was physically elevated away from the cell surface (TR3-DAF), a lack of TRAIL detection was documented in a copy number range where DR5 expression was demonstrated.

In a final attempt to verify these experimental findings, we assessed the expression profiles of GPI-anchored TR3 forms in cells that are DR5-deficient. Chinese hamster ovary cells (CHO), genetically engineered to carry the infectivity-enhancing human Coxsackie-adenovirus receptor [CHO-CAR, ref. 34], were transduced with Ad5-based adenoviral vectors encoding the spacer and non-spacer TR3 constructs (Ad5-TR3-GPI/eY and Ad5-TR3-DAF/eY). Due to the lack of DR5 expression, we anticipated equivalent staining patterns for both membrane-anchored TR3 variants. And indeed, following the same TRAIL detection method as described above, we found that TR3-GPI and TR3-DAF exhibited identical, diagonal staining profiles (Fig. 5d). These results strongly support our hypothesis that the anti-TRAIL mAb binding epitope of spacer-containing TR3-DAF was masked when expressed in DR5-positive cells, but was fully accessible on cells that do not express this (or any other receptor) with affinity to human TRAIL. Conversely, our data are consistent with the notion that membrane-anchored TRAIL forms, such as native TRAIL and spacer-deficient, wild-type-like variants (TR3-GPI and TR3-TM), as well as the mesothelin-tethered soluble TR3 variant SS-TR3, are incapable of interacting with their own receptors when concomitantly present on the same cell membrane.

## Discussion

Targeted cancer drug development offers unique opportunities on the path to higher treatment efficiency and reduced systemic toxicity. In our current study, we have characterized a newly designed variant of the TNF superfamily member TRAIL, designated TR3, targeted to the cancer biomarker mesothelin, using a single chain antibody format (SS-TR3). While we noticed a substantial increase in bioactivity of the targeted therapeutics, the cell death mechanism heavily depended on spatial attributes of the given ligand/receptor pair. It turned out that tethering spacer-deficient SS-TR3 to the mesothelin surface antigen did not eliminate its intended target cells (no evidence for “cis”-acting phenotype). Instead, the SS-TR3-decorated “targets” served as powerful effector cells, killing exclusively via a bystander mechanism (“trans” phenotype). The introduction of a spacer domain, either into the targeted cancer drug itself (SS-S-TR3) or into the target antigen (Meso/DAF), was necessary and sufficient to convert SS-TR3 into a cis-acting cancer drug. These results were unexpected but highly informative as they taught us valuable lessons for the design of biomarker-targeted cancer therapeutics.

Based on the results obtained through functional analyses of membrane-targeted TR3 forms, we hypothesized that, in more general terms, membrane-proximal TR3 variants, possibly including native TRAIL, might not be able to physically engage their own receptors when coexpressed on the same cell membrane. We therefore generated membrane-anchored TR3 variants, with and without spacer, in analogy to their targeted fluid phase counterparts and studied their ability to interact with DR5 on the plasma membrane using flow cytometry. This approach was further inspired by a recent publication in which the authors reported on activated immune effector cells (NK and CD8 + T cells) that express both TRAIL ligand and its receptors (DR5, DcR1 and DcR2) without causing their own cell death^21^. While all membrane-anchored TR3 variants were fully active, we confirmed the phenotypic identity of native TRAIL with membrane-proximal TR3 variants (TR3-GPI and TR3-TM), i.e. the binding epitopes of a blocking anti-TRAIL mAb were permanently detectable even in the presence of DR5 expression and indicate a lack of functional interaction between ligand and receptor. For this interaction to take place, an elongated and flexible linker was required that lifted the TR3 epitope away from the plasma membrane (TR3-DAF), allowing the TR3 epitope to bend over and bind to DR5, leading to a loss of signal detection in a copy number range where DR5 expression was confirmed. This difference in TRAIL surface detection between TR3-GPI (membrane-proximal) and TR3-DAF (membrane-distal) in DR5-positive reporter cells (293T) was completely neutralized when DR5 was not present (CHO).

**Figure 6:**
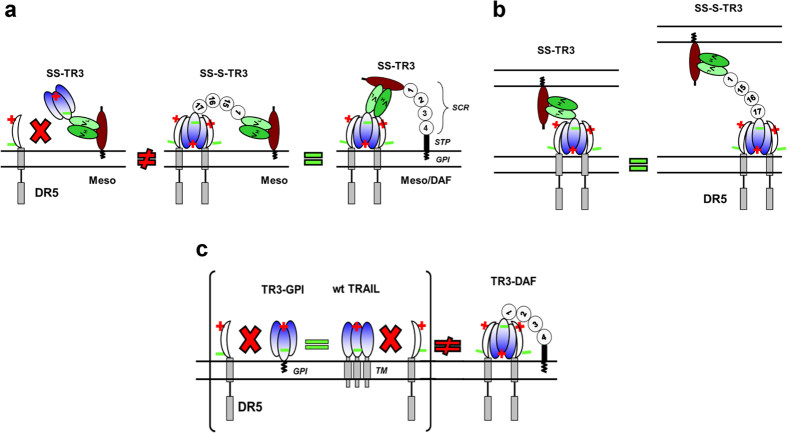
Reciprocal polarities are required for a functional interaction between ligand and receptor of the TNF superfamily member TRAIL. (**a**) Experimental evidence suggests that the mesothelin-targeted SS-TR3 is incapable of inducing apoptosis on cells it binds, represented by a state of “equal polarity” between ligand and receptor. Only an elongated spacer, placed either into the cancer drug itself (SS-S-TR3) or being part of the target antigen (Meso/DAF) that lifts the mesothelin binding site of SS-TR3 away from the membrane, allows formation of a “reciprocal polarity”, a requirement for a functional interaction between ligand and receptors. (**b**) Both mesothelin-targeted TR3 variants are capable of inducing death of bystander cells. (**c**) The phenotypic similarity of native TRAIL with its GPI-anchored variant TR3-GPI prevents apoptosis induction on cells that express both the ligand and the receptor. The “spacer-containing” fusion protein TR3-DAF differs from the latter variants due to the elevated location of the TR3 trimer relative to the plasma membrane. The short consensus repeats (SCRs) of the spacer (SCR1 of DAF and SCRs 15–17 of CR1) as well as DAF (1–4) are indicated for clarity.

The results presented in our current study not only have important implications for the design of recombinant TR3-based cancer therapeutics but also shed new light on the biology of the native TRAIL cytokine. Whenever a TR3-based cancer drug is tethered close to the membrane of its intended target, we propose that under these circumstances, ligand and receptor are facing non-attracting “repulsive forces”, indicated by “equivalent polarities” between TR3 and DR5 (Fig. 6a, left panel). Only a sufficiently long and flexible linker, as part of the cancer drug itself (SS-S-TR3) or incorporated into the mesothelin target antigen (Meso/DAF), facilitates productive physical interactions via attractive “reciprocal polarity” and converts SS-TR3 into a cis-acting biologic (Fig. 6a, center and right panel). These requirements are easily provided for SS-TR3 and SS-S-TR3 when it comes to engaging death receptors located on bystander cells (Fig. 6b), equivalent in nature compared to TRAIL-expressing immune effector cells. Our findings have important implications for choosing the most appropriate cancer drug to meet specific clinical needs. For example, if one was interested in eliminating circulating tumor cells from the periphery, spacer-containing TR3 drugs seem to be the weapon of choice (cis-acting). If one wanted to treat solid tumors, the spacer-deficient drug candidate might be chosen due to its smaller size (better tissue penetrance) and the ability to induce bystander cell death in an overcrowded tumor environment, where tumor cells are located in close proximity to each other. Along these lines, preliminary data do indeed suggest that mesothelin-targeted SS-TR3 outperforms its non-targeted counterpart in a preclinical xenograft model using BxPC3 pancreatic cancer cells implanted subcutaneously into the flanks of nude mice (D. Spitzer, personal communication).

Similar considerations apply to the native cytokine as well as membrane-anchored recombinant TR3 variants. We could demonstrate that spacer-deficient, membrane-anchored TR3 variants were phenotypically identical to native TRAIL, all incapable of engaging their own receptors when coexpressed on the same cell surface (“equivalent polarity” between ligand and receptor), unless a spacer was incorporated into the recombinant TR3 variant (“reciprocal polarity” restored) (Fig. 6c). For example, if one considered cancer cell therapy using patient-derived, genetically engineered effector cells, such as mesenchymal stem cells (MSC) or tumor infiltrating lymphocytes (TIL), one would rather choose the spacer-deficient TR3 variants as they would not trigger short circuit apoptosis signaling and thereby ensuring effector cell survival.

An important role of TRAIL surface expression has been reported for activated NK cells with respect to the prevention of autoimmunity and elimination of transformed host cells^22^,^23^, but the potential for self-elimination caused by death receptor coexpression was not addressed in these studies. We envision a lack of ligand/receptor interaction on the effector cell surface as a requirement for TRAIL to being fully available for an interaction with activating death receptors (DR4 and DR5) on putative target cell membranes, in which the appropriate polarity between ligand and receptor is provided. If TRAIL would be capable of interacting with its own receptors on the same cell membrane, the ligand would be constantly involved in autocrine-like feedback stimulations, causing a risk of unintentional self-elimination of activated NK and CD8 + T cells. Furthermore, a constitutive interaction of membrane TRAIL with its receptor(s) [DR4, DR5 and even surface expressed decoy receptors DcR1 and DcR2, ref. 21], would chronically block the ligand from interacting with receptors located on putative target cells. Our findings might thus not only apply to the native TRAIL ligand/receptor system, but also to other members of this large immune-modulatory cytokine superfamily such as the TNF/TNFR and FasL/Fas system^35^.

In more general terms, we provided experimental evidence that membrane-proximal TRAIL species, including the native cytokine and recombinant, mesothelin-targeted soluble and membrane-anchored TR3 variants, were unable to physically engage with their cognate receptor DR5 when concomitantly expressed on the same cell membrane. Using a set of custom-designed TR3 variants (soluble and membrane-anchored) and a variety of transduction formats (standard transfection methods and adenoviral infection procedures), we connected findings initially discovered during the characterization phase of mesothelin-targeted TR3-based cancer therapeutics with native TRAIL biology. And finally, our results provide important clues for the design of biomarker-targeted TR3-based cancer drugs. According to our data, similar functional (quantitative) properties might be associated with unexpected and undesired qualitative outcomes, which have to be carefully considered for any given ligand/receptor combination.

## Materials and Methods

### Reagents

Recombinant human TRAIL (114281) was purchased from Enzo Life Sciences (Plymouth Meeting, PA). For Western blot analyses, anti-human TRAIL pAb (rabbit) was obtained from Peprotech (Rocky Hill, NJ). Signal detection was achieved with goat anti-rabbit HRP-conjugated secondary antibodies (Santa Cruz Biotechnology, Santa Cruz, CA). A molecular weight marker (Precision Plus Protein Western C Standards and Precision Protein StrepTactin-HRP Conjugate), was obtained from Bio-RAD (Hercules, CA). TRAIL detection for flow cytometry applications was performed using a function-blocking mouse mAb (clone 2E5) purchased from Abcam (Cambridge, MA). Mouse anti-mesothelin mAb (clone K1) was purchased from Santa Cruz Biotech (Santa Cruz, CA). The mouse anti-KDR mAb (clone KDR-1) was purchased from Sigma-Aldrich (St. Louis, MO). Membrane-tethered SS-DAF via flow cytometry was detected with a mouse anti-DAF mAb (clone BRIC216) (Serotech, Oxford, UK). The mouse anti-FLAG mAb (clone M2) was purchased from Sigma-Aldrich (St. Louis, MO). Secondary antibodies for FACS analyses-anti-mouse pAb (IgG) and anti-human pAb (IgG, for the detection of DR5-Fc) (FITC/PE conjugated)-were obtained from Sigma-Aldrich (St. Louis, MO). Anti-FITC MicroBeads were obtained from Miltenyi Biotech (Bergisch Gladbach, Germany). Carboxyfluorescein succinimidyl ester (CFSE) and Z-VAD-FMK were obtained from Enzo Life Sciences (Farmingdale, NY). Custom oligonucleotides were purchased from Integrated DNA Technologies (IDT, Coralville, IA).

### Expression plasmids

The TR3 expression plasmid and the two mouse RBC-targeted variants Ter119-TR3 (no spacer) and Ter119-S-TR3 (containing a chimeric spacer comprised of the human complement regulatory proteins decay-accelerating factor (DAF) [short consensus repeat (SCR1) and complement receptor-1 (CR1) (SCRs 15–17)] have been described earlier^33^. A 738 bp cDNA encoding for the anti-human single chain antibody fragment (scFv) SS [ref. 20] was custom-synthesized (GenScript, Piscataway, NJ). In order to verify its high affinity for human mesothelin, it was subsequently used to replace the corresponding scFv-Ter119 of sT-DAF [ref. 33] with the 738 bp *Bsi*WI scFv-SS fragment, resulting in the mesothelin-targeted form of human DAF (SS-DAF), in which the DAF domain served solely as an epitope tag for immunologic detection purposes. SS-DAF was also used for blocking experiments involving mesothelin-targeted TR3 variants. TR3 variants targeting human mesothelin were generated by replacing the RBC-targeted, spacer-containing and spacer-deficient constructs with the scFv-SS via *Bsi*WI fragment ligation (SS-TR3 and SS-S-TR3). Wild-type TRAIL was generated by PCR using a U937-derived cDNA library [ref. 36] (forward: 5′-GAA TTC CAT GGC TAT GAT GGA GGT CCA GGG-3′; reverse: 5′-TCT AGA TTA GCC AAC TAA AAA GGC CCC G-3′). GPI-anchored TR3 was generated by inserting a 120 bp PCR fragment (forward: 5′-AGA TCT CCA AAT AAA GGA AGT GGA ACC AC-3′; reverse: 5′-TCT AGA TTA AGT CAG CAA GCC CAT GGT TAC-3′) of the C-terminal GPI signal sequence of human DAF into the unique *Bgl*II site at the C-terminus of the TR3 expression plasmid (pTR3-GPIeY). GPI-anchored, spacer-containing TR3-DAFeY (the four SCRs of human DAF serve to elevate the mesothelin portion of the fusion protein away from the plasma membrane) was generated by inserting the 1763 bp *Eco*RI/*Bgl*II (S1-treated) fragment of the TR3 expression plasmid into the 5370 bp *Eco*RI/*Bsi*WI (filled in) plasmid backbone of Ter119-DAFeY [see ref. 33 and D. Spitzer, unpublished data^37^]. The corresponding adenoviral shuttle vectors were established by inserting the respective *Xho*I/*Nhe*I (filled in) fragments of TR3-GPIeY (3380 bp) and TR3DAFeY (4402 bp) into the 7359 bp *Eco*RV/*Xho*I linearized pShuttle-CMV^38^, resulting in pS-TR3-GPIeY and pS-TR3-DAFeY. Signaling-deficient, cytoplasmic tail-truncated human vascular endothelial growth factor receptor-2 (VEGFR-2/KDR) was obtained via RT-PCR [ref. 39] using gene-specific primers (forward: 5′-CTC GAG CCA TGC AGA GCA AGG TGC TGC TGG CC-3′; reverse: 5′-TCT AGA TTA GGC CCG CTT AAC GGT CCG TAG GAT G-3′) according to published methods^33^. The 2387 bp *Xho*I/*Xba*I extracellular KDR fragment, including its transmembrane domain, was subsequently inserted into the 4159 bp *Xho*I/*Xba*I TR3eY plasmid backbone, thereby replacing the TR3 encoding cDNA, resulting in the KDRdCYTeY expression plasmid. In order to generate a transmembrane-anchored TR3 variant, a 165 bp PCR fragment (forward: 5′-AGA TCT GGC GGC GGT GGC TCT GCA AAA GTG GAG GCA TTT TTC ATA ATA G-3′; reverse: 5′-TCT AGA TTA GGC CCG CTT AAC GGT CCG TAG GAT G-3′) of the membrane-spanning domain of human VEGFR-2 was inserted into the 5991 bp TR3 expression plasmid via *Bgl*II/*Xba*I, resulting in pTR3-TMeY. An N-terminal FLAG-tagged, mature mesothelin (GPI-anchored) expression construct was generated by inserting a 1035 bp *Bsi*WI/*Xba*I PCR fragment (forward: 5′-CGT ACG GAC TAC AAG GAC GAT GAT GAC AAA CAG ATC TCC GGT GGA GGC TCA GAA GTG GAG AAG ACA GCC TGT CCT TC-3′; reverse: 5′-TCT AGA TTA GGC CAG GGT GGA GGC TAG GAG CAG-3′) into the 4350 bp *Bsi*WI/*Xba*I-linearized pTR3eY backbone, resulting in the expression plasmid pMeso. For the membrane elevation of the scFv-SS binding site on human mesothelin, the 1454 bp *Eco*RI/*Bgl*II(S1-treated) fragment of GPI-anchor-deleted, FLAG-tagged human mesothelin [ref. 7] was combined with the 5370 bp *Eco*RI/*Bsi*WI(S1-treated) plasmid backbone of Ter119-DAFeY [see ref. 33 and D. Spitzer, unpublished data^37^], resulting in the pMeso/DAF mesothelin/DAF fusion construct. In order to establish Jurkat cell pools stably expressing the mature forms of human mesothelin and the mesothelin/DAF fusion protein, retrovirus-mediated gene transfer was chosen, based on the retroviral vector MFG-eYFP already described in our earlier study [ref. 33]. The 8058 bp *Nco*I (S1-treated)/*Not*I (filled in) MFG-eYFP backbone was combined with the 1163 bp *Eco*RI (filled in)/*Hin*dIII (filled in) fragment of pMeso, resulting in the retroviral vector MFG-Meso. For the construction of the retroviral vector encoding the mesothelin/DAF fusion protein, the 8058 bp *Nco*I (S1-treated)/*Not*I (filled in) MFG-eYFP backbone was ligated with the 2221 bp *Xho*I (filled in)/*Xba*I (filled in) fragment derived from the pMeso/DAF expression plasmid, resulting in the retroviral vector MFG-Meso/DAF. All PCR-derived DNA fragments were verified in the final products by DNA sequencing (Washington University Sequencing Core).

### Cells, transfections and protein production

All cell lines used in the experiments were obtained from the American Type Culture Collection (ATCC, Manassas, VA). Human embryonic kidney cells (HEK293T) were used for protein generation. They were maintained in Dulbecco’s modified Eagle’s medium (DMEM, Invitrogen, Carlsbad, CA) containing 10% fetal calf serum (FCS, Harlan, Madison, WI). Media were supplemented with L-glutamine (Sigma), non-essential amino acids (BioWhittaker, Walkersville, MD) and penicillin and streptomycin (Cellgro, Mediatech, Manassas, VA). The human T cell line Jurkat was maintained in Roswell Park Memorial Institute (RPMI1640) medium (Invitrogen), supplemented with 10% FCS, L-glutamine and penicillin/streptomycin. Human Coxsackie-adenovirus receptor-expressing Chinese hamster ovary cells (CHO-CAR) were maintained in Ham’s F12 medium (Gibco, Life Technologies, Grand Island, NY), supplemented with 10% FCS, L-glutamine and penicillin/streptomycin^34^.

The recombinant TR3 forms and soluble death receptor 5 (DR5-Fc) were prepared by transient expression in HEK293T cells as described^8^ using Opti-Mem serum-free medium (Gibco) and TransIT-293 (Mirus, MIR2700) transfection reagent, as per the manufacturer’s instructions. To obtain concentrated TR3 protein stocks, the supernatants were applied to centrifugal filter devices with a 10 kDa molecular cut-off (Centricon Plus-20, Millipore, Bedford, MD). TR3 protein concentrations were determined via Western blot analysis relative to a known standard (rTRAIL). DR5-Fc was purified using Protein A columns as per the manufacturer’s instructions (Pierce, Rockford, IL). Protein concentration was determined with a spectrophotometer using BSA (New England Biolabs, Ipswich, MA) as a standard.

Generation of recombinant adenovirus. To generate replication incompetent adenovirus (Ad) vectors, the respective shuttle plasmids encoding TR3GPIeY and TR3DAFeY cDNAs were constructed first. Gene expressing in these shuttle vectors is controlled by a strong CMV promoter. They are subsequently used to perform homologous recombination in *E*. coli BJ5183 cells with a pAdEasy-1 plasmid, which contains the complete Ad5 genome devoid only of the E1/E3 genes^40^. The resulting plasmids carrying Ad5-TR3GPIeY and Ad5-TR3DAFeY genomes were validated by PCR, restriction analysis, and partial sequencing, linearized with *Pac*I to release the inverted terminal repeats of the viral genomic DNA and used to transfect HEK293T cells. The rescued Ad5-TR3GPIeY and Ad5-TR3DAFeY vectors were propagated using 911 cells^41^, purified by centrifugation on CsCl gradients according to standard protocols, dialyzed against phosphate-buffered saline (PBS) (8 mM Na_2_HPO_4_, 2 mM KH_2_PO_4_ [pH 7.4], 137 mM NaCl, 2.7 mM KCl]) containing 10% glycerol, and stored at −80 °C until further use. The titers of physical viral particles (v.p.) were determined by methods described by Maizel *et al*. [ref. 42] and calculated as 1.95 × 10^12^/mL for Ad5-TR3GPIeY and 2.25 × 10^12^/mL for Ad5-TR3DAFeY, respectively.

### Immunoblotting

Samples (cell lysates or transfection supernatants) were submitted to 10% SDS-PAGE and transferred onto a nitrocellulose membrane. After blocking with dry milk, the membranes were incubated with the respective primary antibodies (anti-human TRAIL pAb, anti-human mesothelin mAb or anti-FLAG mAb), followed by HRP-conjugated secondary antibodies (anti-rabbit or anti-mouse) and developed with Immunstar Western C kit (Bio-Rad, Hercules, CA) using the Chemidoc XRS plus Imaging system (Bio-Rad).

### Flow Cytometry

Flow cytometry was used to verify the expression pattern of endogenous protein levels (DR5 and DAF), recombinant membrane proteins (Meso, Meso/DAF, and the membrane-anchored TRAIL variants [wt TRAIL, TR3-GPI and TR3-DAF), following transient transfection of HEK293T, stably infection of Jurkat cells (using retroviral vectors) and transient infection of CHO-CAR cells (using adenoviral vectors). CFSE-based cell proliferation assays were performed according to the manufacturer’s recommendations (Sigma-Aldrich, St. Louis, MO). Data acquisition was done on a FACSCalibur flow cytometer (BD Biosciences, San Jose, CA) and data were analyzed using FlowJo software (Version 7.6.5, Tree Star, Ashland, OR).

### Cell proliferation assay

CFSE (5(6)-Carboxyfluorescein diacetate N-hydroxysuccinimidyl ester) was used to monitor the proliferation rate of drug-treated Jurkat cells. A Jurkat cell pool containing ~8% mesothelin-positive Jurkat cells (9 × 10^10^ cells) was stained with 1 μM CFSE at the beginning of the experiment (day 0) according to the manufacturer’s protocol. The cells were then plated in a 24-well plate and treated with 144 pM SS-TR3. Untreated, CFSE-labeled cells were used as a control. During the course of six days, aliquots were removed daily and analyzed via flow cytometry.

### Drug treatment in the presence of apoptosis blockade

Z-VAD-FMK (carbobenzoxy-valyl-alanyl-aspartyl-[O-methyl]fluoromethylketone), a cell-permeant pan-caspase inhibitor, was used to evaluate the expansion profile of mesothelin-positive Jurkat cells following targeted TR3 treatment during apoptosis blockade. A Jurkat cell population containing ~8% mesothelin-positive cells was treated with medium only (control), Z-VAD only (2 μM Z-VAD every day), SS-TR3 and Z-VAD in combination (72 pM SS-TR3 once and 2 μM Z-VAD every day), and SS-TR3 only (72 pM SS-TR3 once). Six days after treatment the cells were stained for mesothelin (via anti-FLAG immunostaining) and analyzed by flow cytometry.

### Animals

C57BL/6 wild type (WT) mice were used as an erythrocyte source as previously described^8^. Blood was collected from the saphenous vein using heparinized glass capillaries for coating experiments with Ter119-(S)-TR3. Procedures involving mice were approved by the Washington University Animal Studies Committee and conducted in accordance with the guidelines for the care and use of laboratory research animals established by the NIH.

### Analysis of cell death

The assay cells were seeded in 96 well plates. Unless otherwise stated, the cells were treated the same day and assayed 18 hours later using CellTiter-Glo Luminescent Viability Assay (Promega, Madison, WI). The luminescence signals were recorded using a Multi-Mode microplate reader (BioTek Instruments, Winooski, VT).

### Statistical analyses

Treatment efficiency of *in vitro* killing assays are presented as means ± SEM. Statistical significance is defined as *P* < 0.05 and was calculated employing analysis of variance (one-way ANOVA, Tukey’s Multiple Comparison Test) and the Student’s *t-test* (unpaired) as indicated using GraphPad Prism (V 6.04) software.

## Additional Information

**How to cite this article**: Tatzel, K. *et al*. Membrane-proximal TRAIL species are incapable of inducing short circuit apoptosis signaling: Implications for drug development and basic cytokine biology. *Sci. Rep.* **6**, 22661; doi: 10.1038/srep22661 (2016).

## Supplementary Material

Supplementary Information
