# Erratum: Membrane-proximal TRAIL species are incapable of inducing short circuit apoptosis signaling: Implications for drug development and basic cytokine biology

**DOI:** 10.1038/srep25217

**Published:** 2016-05-04

**Authors:** Katharina Tatzel, Lindsay Kuroki, Igor Dmitriev, Elena Kashentseva, David T. Curiel, S. Peter Goedegebuure, Matthew A. Powell, David G. Mutch, William G. Hawkins, Dirk Spitzer

Scientific Reports 6: Article number: 2266110.1038/srep22661; published online: 03032016; updated: 05042016

In the Supplementary Information file originally published with this Article, references 32, 33 and 37 were incorrectly given as references 25, 26 and 35 respectively. These errors have been corrected in the Supplementary Information that now accompanies the Article.

