# Supplementary material for: Membrane-proximal TRAIL species are incapable of inducing short circuit apoptosis signaling: Implications for drug development and basic cytokine biology
Source: Sci Rep. 2016 Mar 3;6:22661. doi: 10.1038/srep22661 (PMC4776141; doi:10.1038/srep22661)
Supplement: Supplementary Information [file srep22661-s1.pdf]

Supplementary Information

Membrane-proximal TRAIL species are incapable of inducing short circuit apoptosis signaling: Implications for drug development and basic cytokine biology

Katharina Tatzel[1], Lindsay Kuroki[2], Igor Dmitriev[3], Elena Kashentseva[3], David T. Curiel[3], S. Peter Goedegebuure[1,4], Matthew A. Powell[2,4], David G. Mutch[2,4], William G. Hawkins[1,4] and Dirk Spitzer[1,4]*

[1]Washington University School of Medicine, Department of Surgery, 660 S. Euclid Avenue, St. Louis, Missouri 63110, USA.
[2]Washington University School of Medicine, Division of Gynecologic Oncology, 660 S. Euclid Avenue, St. Louis, Missouri 63110, USA.
[3]Washington University School of Medicine, Department of Radiation Oncology, 660 S. Euclid Avenue, St. Louis, Missouri 63110, USA.
[4]Alvin J. Siteman Cancer Center, 660 S. Euclid Avenue, St. Louis, Missouri 63110, USA.

**Correspondence*
Address correspondence and reprint requests to Dr. Dirk Spitzer, Department of Surgery, Washington University School of Medicine, 660 S. Euclid Avenue, Campus Box 8109, St. Louis, MO 63110, USA, E-mail address: spitzerd@wudosis.wustl.edu

This PDF file includes:

Supplementary Figures S1 – S6

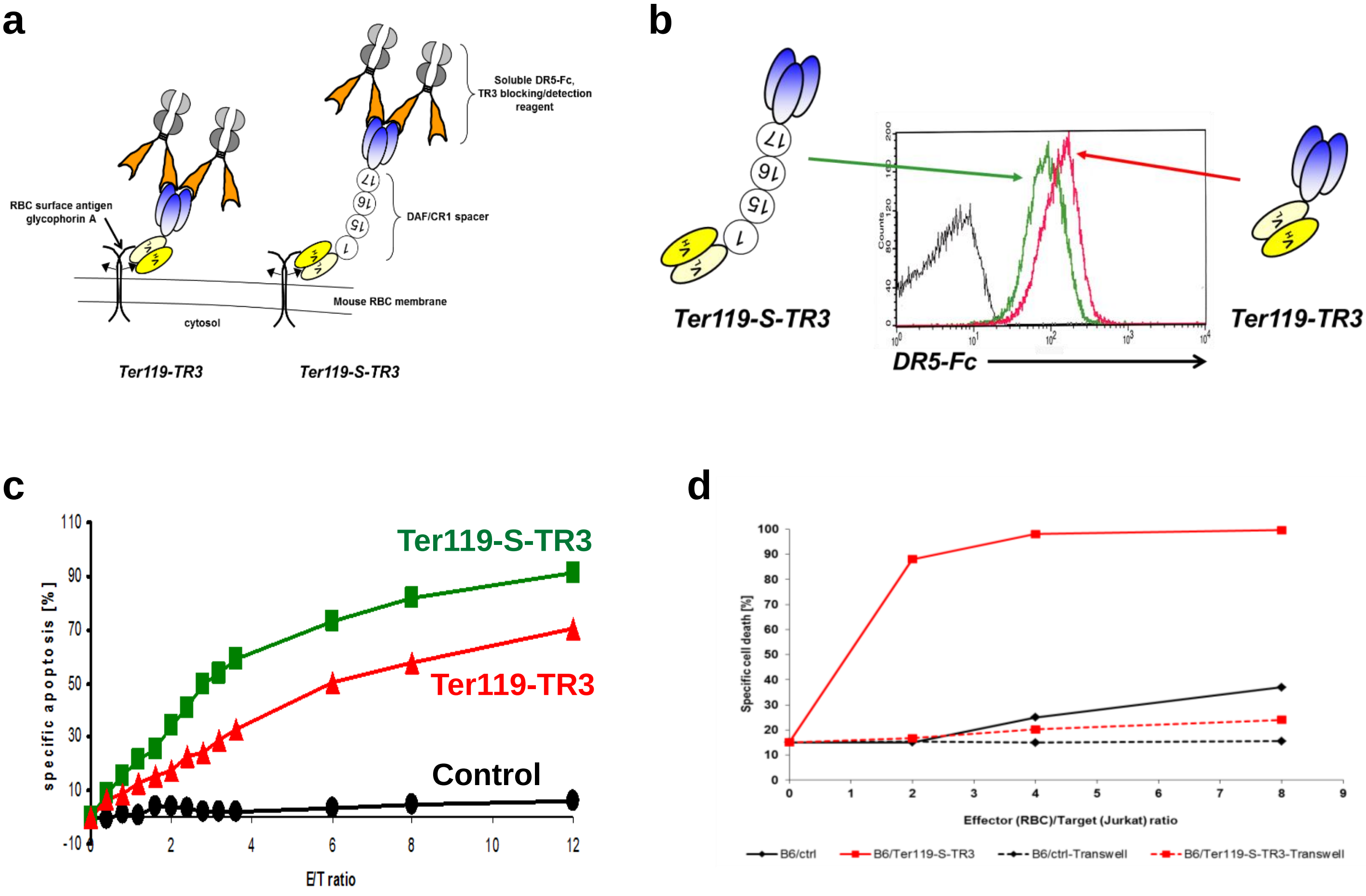

Fig. S1. Characteristic features of the nanoparticle concept based on recombinant TR3 variants tethered to mouse RBCs. (**a**) The scFv-Ter119 [yellow ovals] was fused to the N-terminus of TR3 (blue ovals) without (left, Ter119-TR3) and with an elongated spacer (right, Ter119-S-TR3). The spacer was derived from the globular domains of human CR1 and DAF (see Materials and Methods for details and ref. 33). Immunologic detection on mouse RBCs was performed using soluble DR5-Fc (orange/grey). (**b**) The relative abundance of the spacer-deficient Ter119-TR3 (red line) was ~1.7-fold higher than the spacer-containing Ter119-S-TR3 (green line). (**c**) Functional activity of the same RBC preparations shown in (b) using a coculture assay with human Jurkat cells at increasing effector:target cell ratios (E/T) as described 8. Mouse RBCs incubated with medium alone served as a control. (**d**) Mouse RBCs coated with Ter119-TR3 and Ter119-S-TR3 (compare (b) for coating levels) were treated at increasing effector ratios with Jurkat cells either with (dashed lines) and without transwell separation (solid lines). Non-coated RBCs served as a control. Cell death was determined by FACS analysis as described [8]. Target cell killing was nearly completely abrogated when cell-cell contact was inhibited (dashed red line).

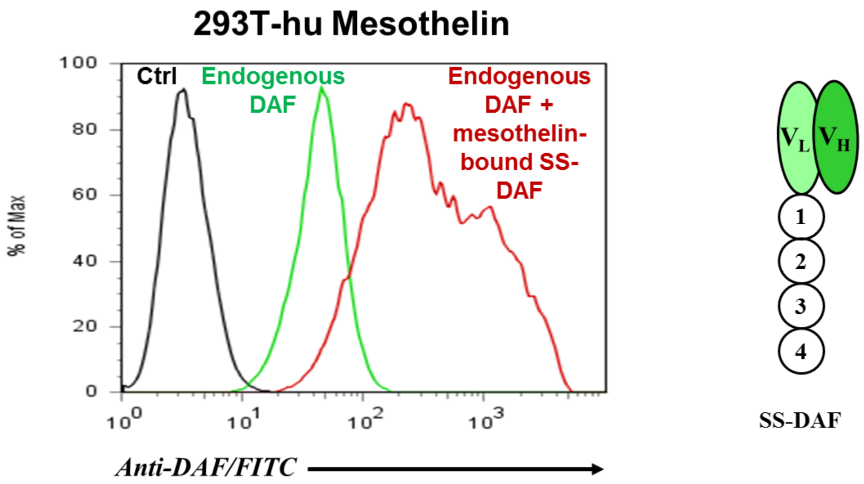

Fig. S2. ScFv-SS facilitates targeting of soluble human DAF to surface-expressed human mesothelin. HEK293T cells were transfected with a full length human mesothelin expression plasmid. ScFv-SS with specificity for human mesothelin [20] was fused to a secreted form of human DAF (SS-DAF, right panel). DAF, comprised of four short consensus repeats (SCRs) was used here solely as an epitope tag for immunologic verification of scFv-SS functionality. Mesothelin-expressing HEK293T cells were incubated with SS-DAF, washed and assessed for scFv-SS binding using a monoclonal Ab against human DAF and subjected to FACS analysis. Black line: secondary Ab only on SS-DAF-treated mesothelin-expressing HEK293T cells (control), Green line: endogenous DAF level, Red line: combined staining profile of endogenous and exogenous DAF attached to the target cells via mesothelin binding.

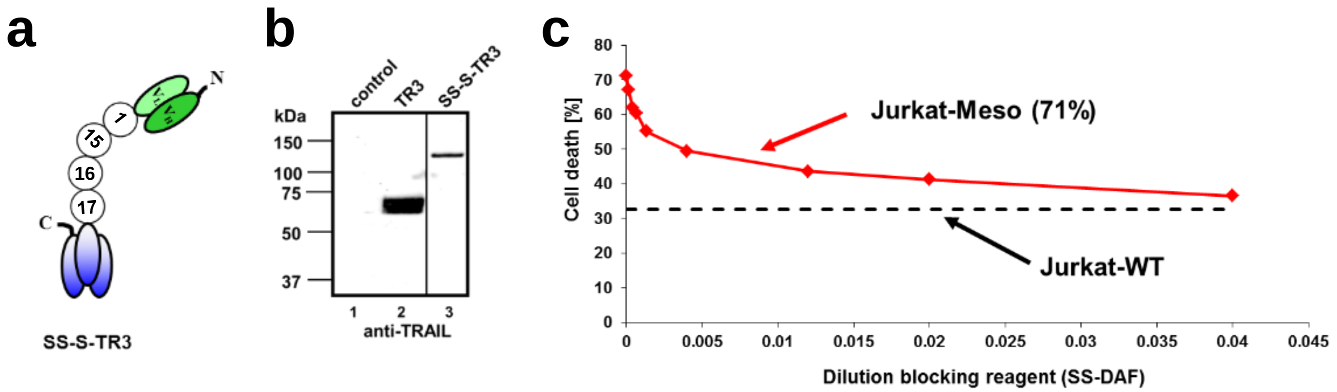

Fig. S3. Robust increase in killing capacity of ectopically mesothelin-expressing Jurkat tumor cells. (**a**) Schematic representation of spacer-containing SS-S-TR3. The spacer is comprised of the globular domains of human decay-accelerating factor (DAF) and complement regulator 1 (CR1)[33]. (**b**) Western blot analysis confirms the molecular weight of SS-S-TR3 (120 kDa) relative to TR3 (61 kDa). (**c**) Stable mesothelin-expressing Jurkat cells (Jurkat-Meso, 71%) were challenged with SS-S-TR3 (>70% cell death in the absence of SS-DAF) in the presence of increasing amounts of SS-DAF, leading to a progressively reduced killing capacity of SS-S-TR3. At the highest SS-DAF concentration (dilution 0.04), target cell killing of the Jurkat-Meso cell pool reached that of the killing capacity of SS-S-TR3 on Jurkat-WT cells (dashed black line). Cell death was determined by FACS analysis as described[33].

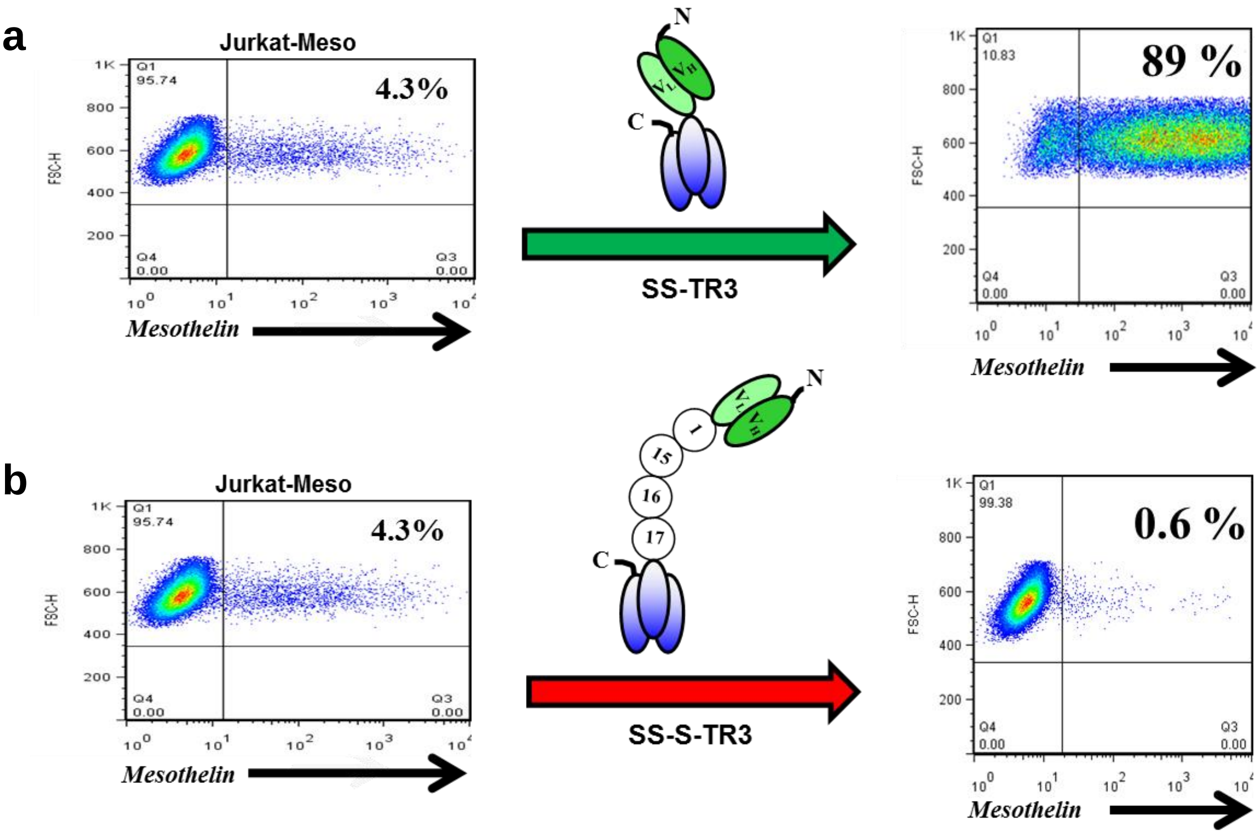

Fig. S4. The spacer influences the nature of mesothelin-targeted TR3-mediated tumor cell killing. A stably mesothelin-expressing Jurkat cell pool (Jurkat-Meso, ~4%), was treated with equipotent concentrations of SS-TR3 (**a**) and SS-S-TR3 (**b**) (for comparison see TR3 and SS-TR3 activity profiles on Jurkat-WT cells in Fig. 1e [SS-S-TR3, not shown and ref. 37]). Following a six day recovery phase, changes in the ratio of Jurkat-Meso cells were monitored by flow cytometry. Besides similar increases in overall killing capacities of targeted SS-TR3 and SS-S-TR3 [not shown and ref. 37], treatment with SS-TR3 resulted in an increase in the ratio of Jurkat-Meso cells from 4% to nearly 90%. Treatment with SS-S-TR3 resulted in a decrease in the ratio of Jurkat-Meso cells from 4% to less than 1%.

a

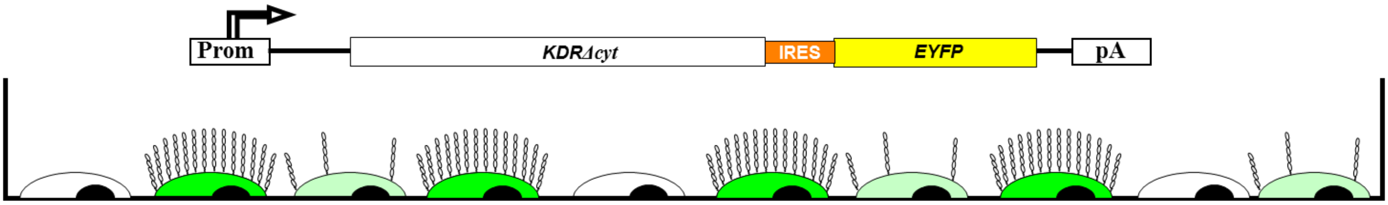

b

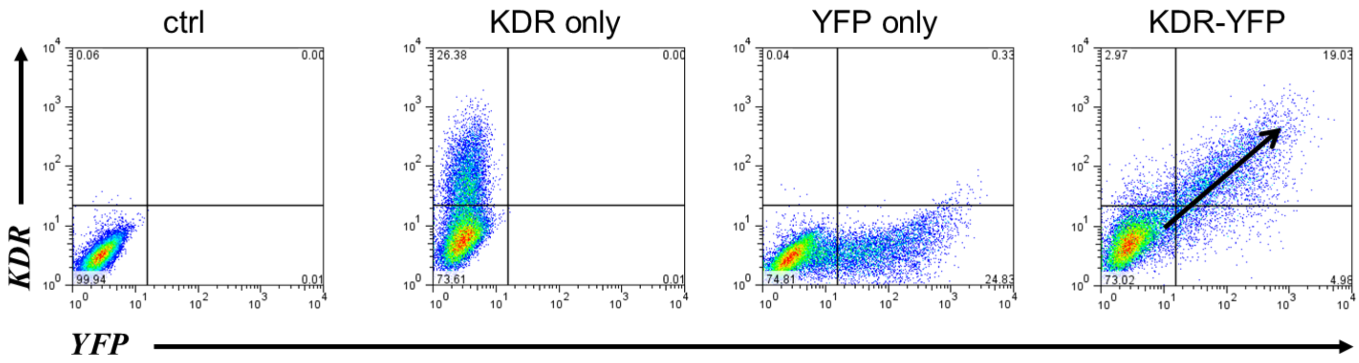

Fig. S5. Bicistronic expression configuration as a means to study ligand/receptor interactions at the plasma membrane. (**a**) Schematic illustration of a bicistronic expression format[32], in which the expression level of a membrane-anchored surface molecule is proportionally correlated with the intensity of the marker protein (eYFP). (**b**) HEK293T cells were transiently transfected with a recombinant, signaling-deficient form of human vascular endothelial growth factor receptor-2 (VEGFR-2, KDRdCYT-eYFP). The cells were stained the following day with an anti-KDR mAb and submitted to FACS analysis. The resulting expression pattern is characteristic for a proportional relationship between the YFP marker and the surface receptor (KDR-YFP, diagonal arrow), which is maintained over the entire expression range and documents unrestricted accessibility of the detection reagent to its binding epitope of the surface antigen.

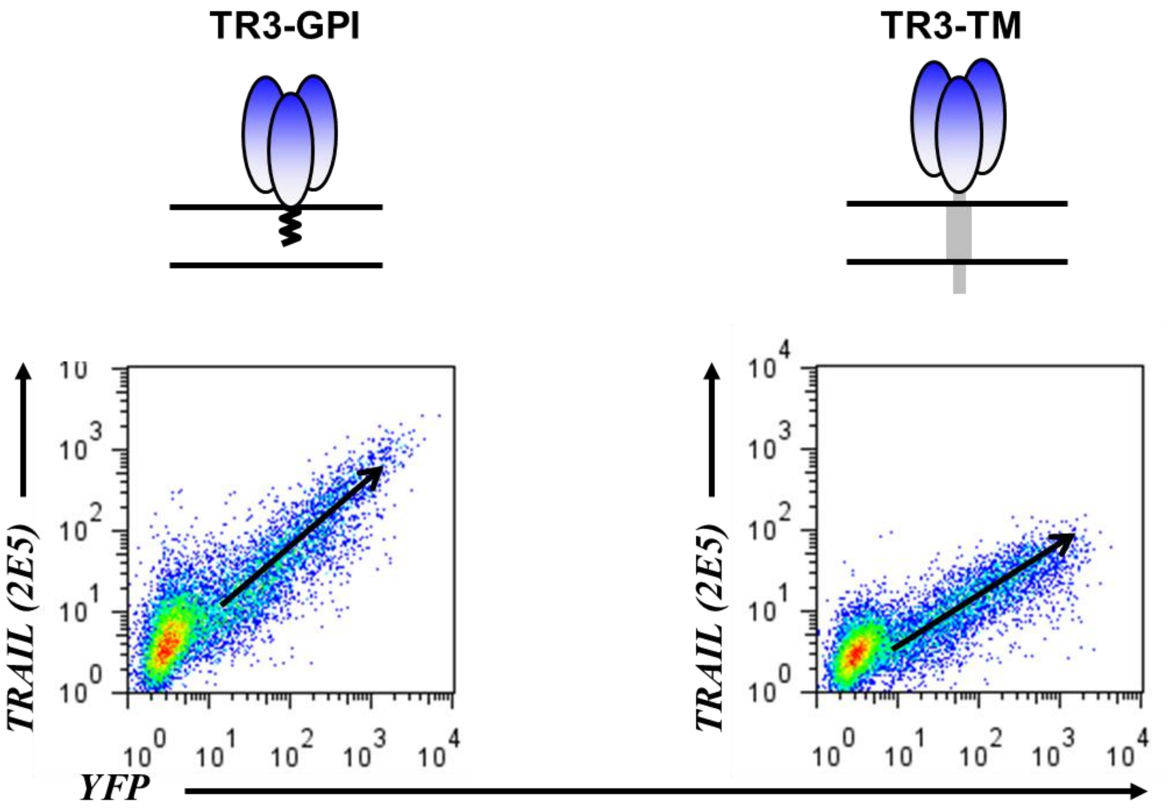

Fig. S6. Transmembrane and GPI-anchored TR3 variants display equivalent staining profiles in DR5-expressing mammalian cells. In order to assess the phenotypic characteristics of membrane anchorage of recombinant TR3, DR5-positive HEK293T cells were transiently transfected with the bicistronic expression plasmids TR3-GPI and TR3-TM (compare Fig. S5 for specifics about plasmid design). The next day, the cells were prepared for flow cytometry using the blocking anti-TRAIL detection antibody 2E5. The primary antibody was visualized with a PE-conjugated secondary antibody. Please note that the expression profiles of TR3-GPI and TR3-TM are equivalent and result in a diagonal pattern (proportional relationship between TR3 and the YFP marker), similar to the expression profile of wt TRAIL (Fig. 5c).
